# Proportion and clinical characteristics of keratoconus among patients attending refraction units: a retrospective facility-based study in Malawi

**DOI:** 10.1007/s10792-026-04161-0

**Published:** 2026-07-15

**Authors:** Leaveson Thom, Vanessa V. R. Moodley

**Affiliations:** 1 Discipline of Optometry, University of KwaZulu Natal, Durban, South Africa; 2 Keratoconus Foundation, Durban, South Africa

**Keywords:** Keratoconus, Corneal ectasia, Facility based proportion, Visual impairment, Refractive errors, Malawi

## Abstract

**Purpose:**

This study assessed the facility-based proportion, demographic profile, clinical characteristics, and factors associated with KC among patients attending tertiary referral refraction clinics in Malawi.

**Methods:**

A retrospective cross-sectional review of clinical records was conducted at Malawi’s four national referral eye hospitals. Records of patients aged 10–45 years who attended refraction services between January 2014 and December 2018 were reviewed. KC cases were ascertained using predefined clinical criteria. Analyses were performed using R version 4.5.1 (R Foundation for Statistical Computing).

**Results:**

Of the 1,400 targeted records, 1,180 (84.3%) were successfully retrieved. The facility-based proportion of KC was 5.7% (67/1180; 95% CI: 4.4–7.2%). KC was most common among individuals aged 18–35 years (*p* = 0.001) and varied significantly by facility and region (*p* < 0.005). Blurred vision was the predominant presenting complaint (88.1%), while diagnosis relied primarily on retinoscopy, slit-lamp examination, and keratometry. Significant inter-eye differences were observed for best-corrected visual acuity and astigmatic axis (*p* < 0.005), but not for keratometric measurements. Median visual acuity improved from 0.2 to 0.6 following refractive correction. In multivariable analyses, cylindrical refractive error remained independently associated (AUC ≈ 0.88) with KC, whereas age and gender were not. Sensitivity analyses produced findings consistent with the primary results.

**Conclusion:**

Keratoconus represents a substantial clinical burden within Malawi’s tertiary eye-care system and is associated with considerable visual impairment. Strengthening early detection, access to corneal imaging, specialised rehabilitation services, and clinical information systems may improve diagnosis, management, and visual outcomes.

## Introduction

Keratoconus (KC) is a progressive ectatic corneal disorder characterized by bilateral but often asymmetric corneal thinning and protrusion, resulting in irregular astigmatism, myopia, and progressive visual impairment. The disease typically affects the central or paracentral cornea, particularly the inferotemporal region, and usually manifests during adolescence or early adulthood, although cases in younger children have increasingly been reported [1]. The aetiology of KC is multifactorial and involves complex interactions between genetic, environmental, and behavioural factors. Several risk factors have been associated with the disease, including eye rubbing, atopy, ultraviolet radiation exposure, family history, connective tissue disorders, allergic diseases, sleeping position, and parental consanguinity [1–5].

The reported occurrence of KC varies considerably across populations and geographical regions owing to differences in diagnostic criteria, disease classification systems, study populations, ethnicity, age distribution, and availability of diagnostic technologies [6–10]. Advances in corneal imaging, particularly corneal topography and tomography, have facilitated earlier and more accurate detection of KC, leading to higher reported frequencies than the historically accepted prevalence of approximately 1 in 2,000 individuals [11–14]. For instance, a systematic review of African studies estimated a facility-based occurernce of 7.9% (95% CI: 2.5–16.0%), with a higher distribution reported among males than females [13].

Early KC may remain asymptomatic and therefore undetected, while progressive visual blurring is the most commonly reported symptom as the disease advances [15, 16]. Diagnosis can be established through routine clinical examinations, including visual acuity assessment, refraction, retinoscopy, slit-lamp biomicroscopy, and keratometry, with advanced investigations such as corneal topography and tomography providing more detailed assessment and disease staging [11]. Severity can be graded using established classification systems, including the Amsler–Krumeich, Collaborative Longitudinal Evaluation of Keratoconus (CLEK), and Belin ABCD classifications [17–19]. Management varies according to disease severity and includes spectacles, contact lenses, corneal cross-linking, and surgical interventions in advanced cases [14, 15, 20–23]. However, despite growing recognition of KC as an important cause of visual impairment and increasing reports from other regions, data on its burden and clinical profile in Malawi remain unavailable. This study therefore assessed the facility-based proportion of keratoconus and its demographic, visual, and refractive characteristics among patients attending refractive units at the four major referral eye hospitals in Malawi. The findings will provide evidence to support clinical service planning, resource allocation, and the development of a multi-disciplinary, multi-sectoral national KC management guidelines.

## Materials and methods

This study used a quantitative approach, applying a cross-sectional, descriptive, retrospective chart review design to obtain the facility-based proportions, demographic and clinical profiles of keratoconus in Malawi.

### Study setting

The study was conducted at public health facilities in Malawi, with specific study sites being the four central hospitals, which are the main referral facilities in their respective regions (Southern, Central and Northern). All central hospitals have an eye department and/or unit with a refraction section. Gatekeeper permission was sought from all facilities prior to the data collection.

### Study population

The study included clinical records of all patients aged 10–45 years who attended the refraction units of the four central hospitals between January 2014 and December 2018. The age range of 10–45 years was selected because keratoconus typically manifests and progresses during adolescence and early adulthood, while diagnoses outside this range are less common and may represent atypical disease patterns. These hospitals serve as the main ophthalmic referral centres in the Southern, Central, and Northern regions of Malawi.

### Study sample, case definition and ascertainment

A minimum sample size of 1400 records was estimated using Cochran’s formula (n = [Z^2^ x p x (1-p)] ÷ d^2^). However, because the study aimed to include all available records, a retrospective census approach was adopted for the 1180 records (84%) which were successfully retrieved due to incomplete archival systems across facilities.

All retrieved records were systematically screened using predefined inclusion and exclusion criteria. Records were excluded if they contained a history of ocular surgery, significant coexisting ocular pathology, missing key clinical information (including visual acuity or keratometry readings), duplicate entries, or if patients were outside the age range of 10–45 years.Because the original diagnostic criteria were not explicitly documented, available clinical findings recorded in the charts including steep keratometry values, irregular keratometric mires, scissors reflex, Charleux oil droplet sign, Vogt’s striae, and Fleischer’s ring—were reviewed to support retrospective case ascertainment. The absence of corneal pachymetry and topography/tomography results from all the files led to heavy reliance on the steepest keratometry (K_s_) values which was computed for each participant. Ks was used only for case ascertainment and severity classification and was not included as a predictor in regression models. Only definite and probable KC were included in the primary data as KC cases. Detailed keratoconus case definition was as per Table 1 below.

**Table 1:** Case definition of keratoconus from the clinical record cards

Classification	Definition	Available evidence from patient record
Definite keratoconus	High diagnostic certainty based on documented clinical diagnosis supported by objective clinical findings	Recorded diagnosis of keratoconus and at least one of the following: (i) abnormal keratometry consistent with KC (e.g., steep K values ≥ 45 D, marked asymmetry, or irregular corneal curvature), (ii) documented severity grading (mild, moderate, severe, advanced), (iii) referral for corneal cross-linking, corneal specialist assessment, or corneal transplantation
Probable keratoconus	Moderate diagnostic certainty based on specialist diagnosis without complete objective documentation	Recorded diagnosis of keratoconus in the patient record but without either documented keratometric findings, severity grading, or advanced management indicators. Some diagnosed KC records lacked numeric keratometry values but were retained based on other slit lamp findings
Keratoconus suspect	Features suggestive of keratoconus but insufficient evidence for confirmed disease	Diagnosis documented as "keratoconus suspect", "KC suspect", or equivalent terminology, with or without abnormal refractive findings or keratometry
Possible keratoconus	Clinical suspicion based on examination findings but no explicit diagnosis	Irregular astigmatism, reduced best-corrected visual acuity not explained by other pathology, abnormal retinoscopy findings, or steep keratometry suggestive of KC without documented diagnosis
Excluded	Insufficient evidence for keratoconus or alternative diagnosis documented	Records with no KC diagnosis, no suggestive clinical findings, or diagnosis attributable to another ocular condition

All records were reviewed using the same criteria to ensure uniform case ascertainment across all facilities. Final facility distribution included 350 records each from Queen Elizabeth Central Hospital (QECH) and Mzuzu Central Hospital (MCH), 264 from Kamuzu Central Hospital (KCH), and 216 from Zomba Central Hospital (ZCH). Relevant demographic, clinical, and management data were extracted using a standardized data collection tool and entered into Microsoft Excel for analysis.

### Ethical considerations

Ethical clearance to conduct this study was obtained from UKZN’s Humanities and Social Sciences Research Ethics Committee **(HSSREC/00008212/2025)** and the Malawi’s National Committee on Research in the Social Sciences and Humanities **(NO. P.10/24/926).** All procedures adhered to the ethical standards and guidelines of the Helsinki Declaration involving human subjects. To ensure de-identification of patients, all record forms were anonymized with codes used instead of names. The data was reported in an aggregated form, no single case was described.

### Data analysis

All analyses were conducted using R version 4.5.1 (R Foundation for Statistical Computing, Vienna, Austria). To enable easy primary analysis, Keratoconus severity was categorized using steepest keratometry (K_s_) based severity thresholds adapted from commonly used keratoconus severity classifications. Although these thresholds broadly reflect increasing disease severity, they do not represent the full original Collaborative Longitudinal Evaluation of Keratoconus (CLEK) classification system. The severity was as follows; Normal/Suspect/Subclinical (Ks < 45.0 D), Mild Stage (45.0 ≤ Ks < 48.0 D), Moderate Stage (48.0 ≤ Ks < 52.0 D), Severe (Ks ≥ 52.0 D). For regression analyses, KC was dichotomized as definite /probable KC (Stages Mild–Severe) versus normal/suspected/possible KC (Table 1). Continuous variables were summarized as medians (interquartile range, IQR) and categorical variables as frequencies and percentages. Between-group differences were assessed using the Wilcoxon rank-sum test for continuous data and the Chi-square or Fisher’s exact test for categorical variables. Statistical significance was set at p < 0.05. To evaluate whether visual impairment severity differed by age and gender, ordinal logistic regression analyses were performed using WHO visual impairment categories as the ordinal outcome variable. Separate models were fitted for uncorrected distance visual acuity (UCDVA) and best-corrected distance visual acuity (BCDVA). Statistical significance was set at *p* < 0.05.

### Regression analysis

Variables associated with keratoconus at *p* < 0.05 in bivariate analysis were considered for inclusion in multivariable logistic regression. In addition, age group and gender were included a priori as potential confounders due to their known influence on the epidemiology and refractive characteristics of keratoconus in previous studies. Variables were selected based on clinical relevance and statistical significance in bivariate analyses; Model A included age group, gender, region and left-eye cylinder power (OS), and Model B replaced region with facility to assess potential clustering effects by site. OS was included in the multivariable models as it demonstrated a stronger and more stable association with keratoconus than OD (right-eye) measurements. Right-eye cylindrical power (OD) was excluded after model refinement. Adjusted odds ratios (aORs) with 95% confidence intervals (CIs) were reported. Model discrimination was evaluated using the area under the receiver operating characteristic curve (AUC), and adjusted marginal probabilities were computed using the R packages.

To assess the adequacy of the sample size for multivariable logistic regression, events-per-variable (EPV) calculations were performed for all candidate models using the smaller outcome category (keratoconus cases). Model stability was assessed against commonly recommended EPV thresholds of 5 and 10, with values ≥ 10 considered desirable and values between 5 and 10 interpreted with caution due to an increased risk of overfitting. Because the number of keratoconus cases was relatively small and some categories contained sparse data, a sensitivity analysis using Firth penalized logistic regression was performed to assess the potential influence of small-sample and sparse-data bias on parameter estimates. Penalized models were fitted using the same covariates as the primary multivariable logistic regression models. Results were compared with conventional maximum-likelihood logistic regression estimates to evaluate the robustness of the observed associations.

### Sensitivity analysis

For sensitivity analyses, a more conservative case definition based on strong clinical evidence was applied (Table 2), while records with incomplete objective or staging evidence were treated as diagnostically uncertain. A sensitivity analysis compared Model A (region-adjusted) and Model B (facility-adjusted) to evaluate the robustness of geographic and site-level effects. Differences in effect sizes and AUCs were used to assess consistency across models.

**Table 2:** Operational case definitions used in the sensitivity analysis

Case category	Operational definition	Used for analysis
Main KC case	Record where diagnosis text indicates keratoconus after cleaning spelling/case variants	Main descriptive case count and profile
Strong clinical evidence KC case	Main KC case with numeric K_s_ (Steepest K reading) recorded for at least one eye and documented KC stage/classification in OD, OS or OU	Sensitivity analysis. This is the conservative KC case definition
KC diagnosis with incomplete objective/staging evidence	Diagnosis indicates KC, but numeric Ks (steepest K reading) and/or stage/classification evidence is incomplete	Reported separately to show diagnostic uncertainty and potential effect on the reported proportion
Not KC/other	Diagnosis text does not indicate keratoconus after cleaning	Excluded from KC case analysis

### Data visualization

Data visualization was performed using the ggplot2 and MASS packages. Box and whisker plot illustrated the distribution of KC stages by age and gender. Visual acuity (VA) was analysed as uncorrected (UCDVA) and best-corrected (BCDVA), categorized by WHO visual impairment thresholds (No impairment, mild, moderate, severe and blindness). For easy analysis, all Snellen visual acuity were converted to decimal notations. Faceted scatter plot compared visual acuity severity by age, gender and between eyes (OD vs OS). All analyses were reproducible, standardized, and graphically harmonized for comparability.

## Results

### Participant characteristics and proportion of keratoconus

Out of the 1,180 patient records which were reviewed, 52.2% (616/1180) of participants were females, while males accounted for 47.8% (564/1180). Participants were recruited from four tertiary referral hospitals, with the largest contributions from MCH and QECH (350 records each). The facility-based proportion of keratoconus was 5.7% (67/1180; 95% CI 4.4–7.2). Proportion varied significantly by age group, facility, region and ethnicity, with the highest proportions observed among individuals aged 18–35 years and at ZCH, whereas no significant gender differences were observed (Table 3).

**Table 3:** Proportion of keratoconus by participant characteristics

	Total (n)	Keratoconus (n)	Proportion (%, 95% CI)	*p*-value
Overall	1180	67	5.7 [4.4, 7.2]	
*Gender*				
female	616	34	5.5 [3.9, 7.6]	0.806
male	564	33	5.9 [4.1, 8.1]	
*Age group (years)*				
Under 18	228	17	7.5 [4.4, 11.7]	0.001
18–35	366	35	9.6 [6.8, 13.0]	
36–45	577	15	2.6 [1.5, 4.3]	
*Facility*				
KCH	264	17	6.4 [3.8, 10.1]	0.002
MCH	350	9	2.6 [1.2, 4.8]	
QECH	350	19	5.4 [3.3, 8.3]	
ZCH	216	22	10.2 [6.5, 15.0]	
*Region*				
Central	264	17	6.4 [3.8, 10.1]	0.010
Northern	350	9	2.6 [1.2, 4.8]	
Southern	566	41	7.2 [5.2, 9.7]	
*Ethnicity*				
African	1127	64	5.7 [4.4, 7.2]	0.019
Asian	46	1	2.2 [0.1, 11.5]	
Caucasian	7	2	28.6 [3.7, 71.0]	

Interpretation should be cautious due to the very small number of non-African participants resulting in extremely wide confidence intervals

KCH^*^ Kamuzu Central Hospital, MCH^*^ Mzuzu Central Hospital, QECH^*^ Queen Elizabeth Central hospital, ZCH^*^ Zomba Central Hospital

### Clinical characteristics and management

Blurred vision was the predominant presenting complaint. Diagnosis relied mainly on retinoscopy, slit-lamp examination and keratometry, while advanced imaging modalities were rarely used. With-the-rule astigmatism was the most common pattern, and spectacles with or without referral for CXL constituted the predominant management approaches (Table 4).

**Table 4:** Clinical characteristics management of participants with confirmed keratoconus (N = 67)

Characteristic	n (%) or Median (IQR)
*Chief complaint*	
Blurred/reduced vision only	59 (88.1)
Itchiness/ocular irritation	5 (7.5)
Diplopia	1 (1.5)
Other complaints	2 (3.0)
*Diagnostic tool used*	
Retinoscope and keratometer	17 (25.4)
Retinoscope, slit lamp, keratometer	49 (73.1)
Retinoscope, slit lamp, keratometer, pachymeter	1 (1.5)
*Type of astigmatism*	
With-the-rule	30 (44.8)
Against-the-rule	18 (26.9)
Oblique	8 (11.9)
Mixed/combined patterns	11 (16.4)
*Clinical management*	
Spectacles only	33 (49.3)
Soft Contact lenses only	3 (4.5)
Spectacles and RGP contact lenses	4 (6.0)
Eye drops only	1 (1.5)
Spectacles and eye drops	2 (3.0)
Spectacles and referral for CXL	24 (35.8)

OCT^*^ Optical Coherence Tomography, CXL^*^ Corneal cross-linking

### Comparison of right and left eye measurements

Inter-eye comparisons demonstrated significant differences only for BCDVA (*p*-value = 0.015) and axis (*P*-value = 0.016), while keratometry, UCDVA, and refractive components showed no statistically significant inter-eye differences (Table 5).

**Table 5:** Paired comparison of right and left eye measurements among participants with confirmed keratoconus

Characteristic	Equal, n (%)	Left worse, n (%)	Right worse, n (%)	*p*-value
*Keratometry (dioptres)*				
Steep K	1 (1.8)	29 (50.9)	27 (47.4)	0.242
Flat K	1 (2.6)	16 (42.1)	21 (55.3)	0.613
*Visual acuity (decimal)*				
BCDVA	35 (52.2)	23 (34.3)	9 (13.4)	0.015
UCDVA	36 (53.7)	18 (26.9)	13 (19.4)	0.511
*Refraction (dioptres)*				
Spherical component	15 (23.8)	28 (44.4)	20 (31.7)	0.178
Cylindrical component	9 (14.5)	24 (38.7)	29 (46.8)	0.345
*Axis (degrees)*				
Axis	23 (37.1)	26 (41.9)	13 (21.0)	0.016

Data are presented as n (%). “Worse” eye was defined based on higher values for keratometry and refractive measures. For visual acuity, lower values indicate worse vision. P-values were derived using the Wilcoxon signed-rank test

UCDVA^*^ uncorrected distance visual acuity, BCDVA^*^ Best corrected distance visual acuity

### Distribution of keratoconus severity by age and gender (sensitivity analysis)

**Fig. 1:**
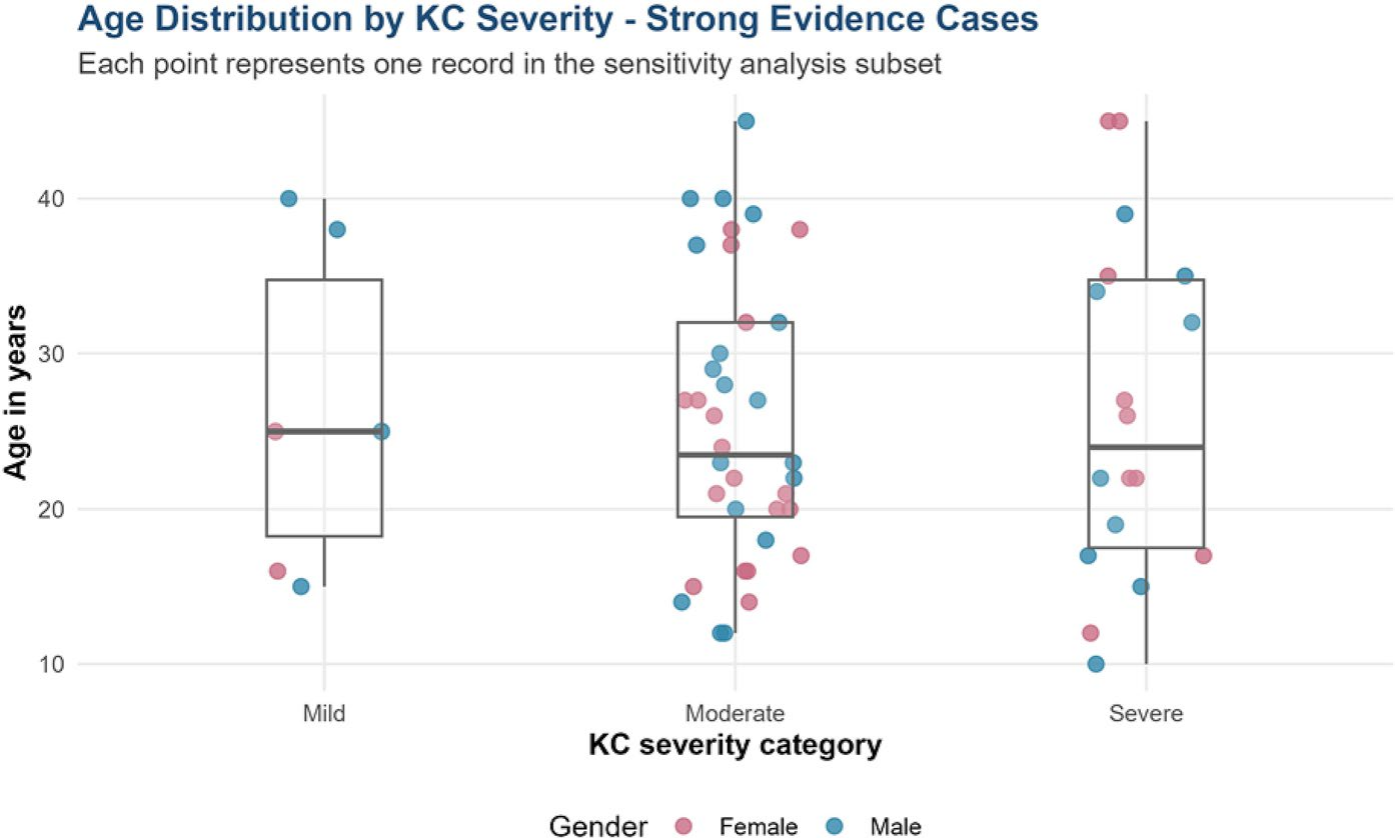
Keratoconus severity by age and gender

In the sensitivity-analysis cohort (Fig. 1), age distributions were broadly similar across mild, moderate, and severe keratoconus categories. Median ages were 24 years (IQR 19–31), 25 years (IQR 20–33), and 26 years (IQR 21–34) for mild, moderate, and severe keratoconus, respectively. Moderate and severe keratoconus cases were observed across a wide age spectrum, and visual inspection of the distributions did not suggest a clear trend of increasing severity with age. Male and female participants were represented throughout all severity categories.

### Distribution of flat and steep average keratometry readings

**Figures 2:**
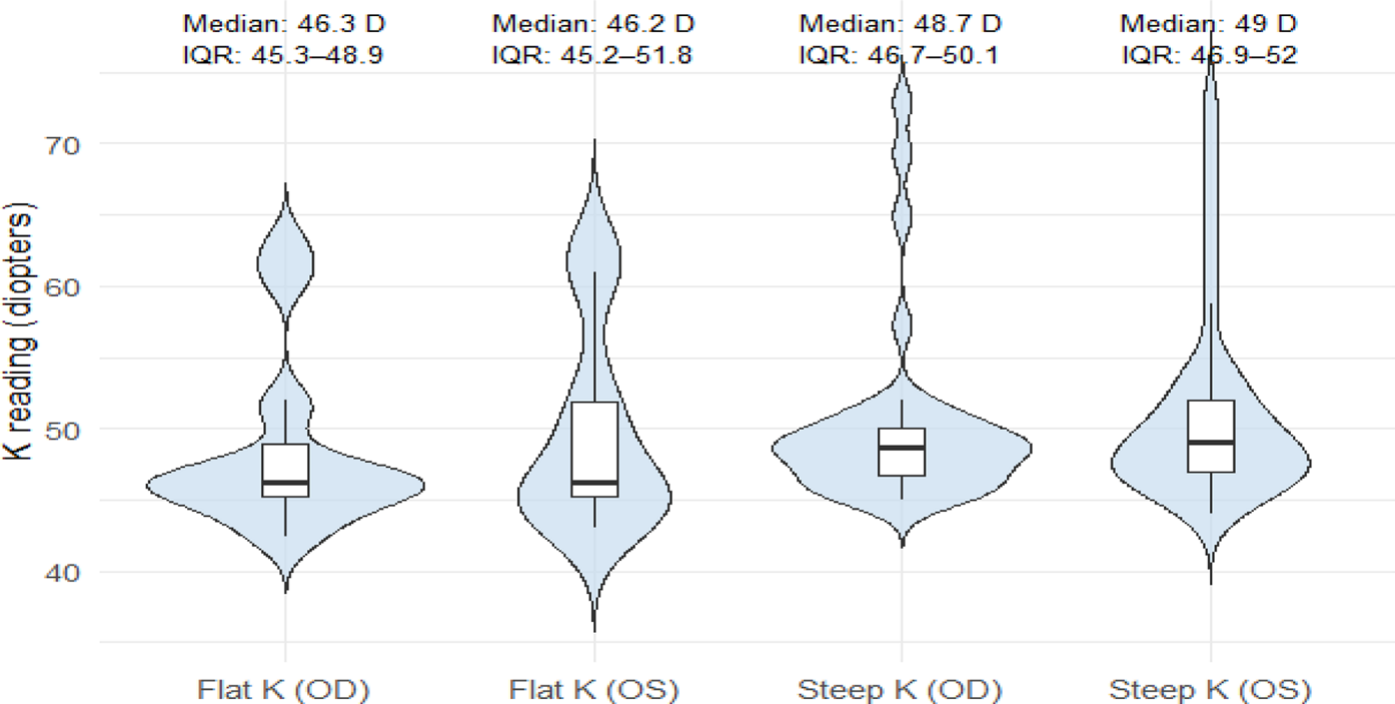
Distribution of flat and steep keratometry readings. *Key* OD* = Oculus dexter (Right eye), OS* = oculus Sinister (left eye) UCDVA* = Uncorrected distance visual acuity, BCDVA* = Best corrected distance visual acuity

Median flat keratometry readings were similar between eyes, measuring 46.3 D in the right eye (OD; IQR 45.3–48.9) and 46.2 D in the left eye (OS; IQR 45.2–51.8). In contrast, steep keratometry readings were higher, with a median of 49.0 D in the left eye (OS; IQR 46.9–52.0) and 48.7 D in the right eye (OD; IQR 46.7–50.1) (Fig. 2).

#### Ks in OD vs Ks in OS scatter plot

**Fig. 3:**
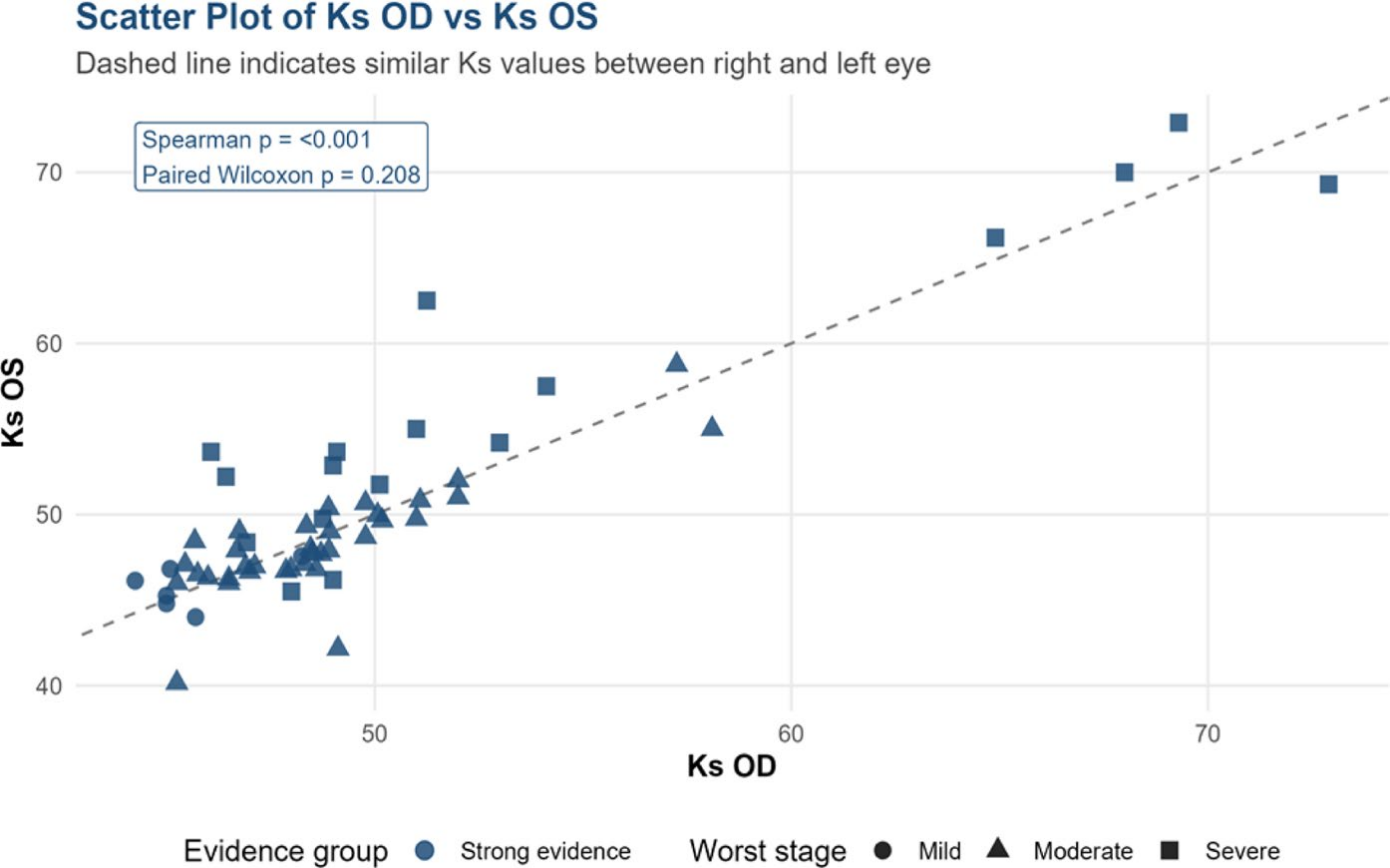
Ks OD vs Ks OS Scatter Plot. *Key* Ks^*^ = Steep Keratometry reading, OD* = Oculus dexter (Right eye), OS* = oculus Sinister (left eye)

Ks values were strongly correlated between the right and left eyes (Spearman’s correlation was statistically significant (*p* < 0.001)). Although several patients demonstrated inter-eye asymmetry, particularly in severe keratoconus, the paired Wilcoxon test showed no significant difference in median Ks values between eyes (*p* = 0.208). Most cases clustered near the line of equality, suggesting generally symmetrical corneal steepening between fellow eyes (Fig. 3).

### Distribution of uncorrected and best-corrected distance visual acuity severity by age, gender and eye

**Fig. 4:**
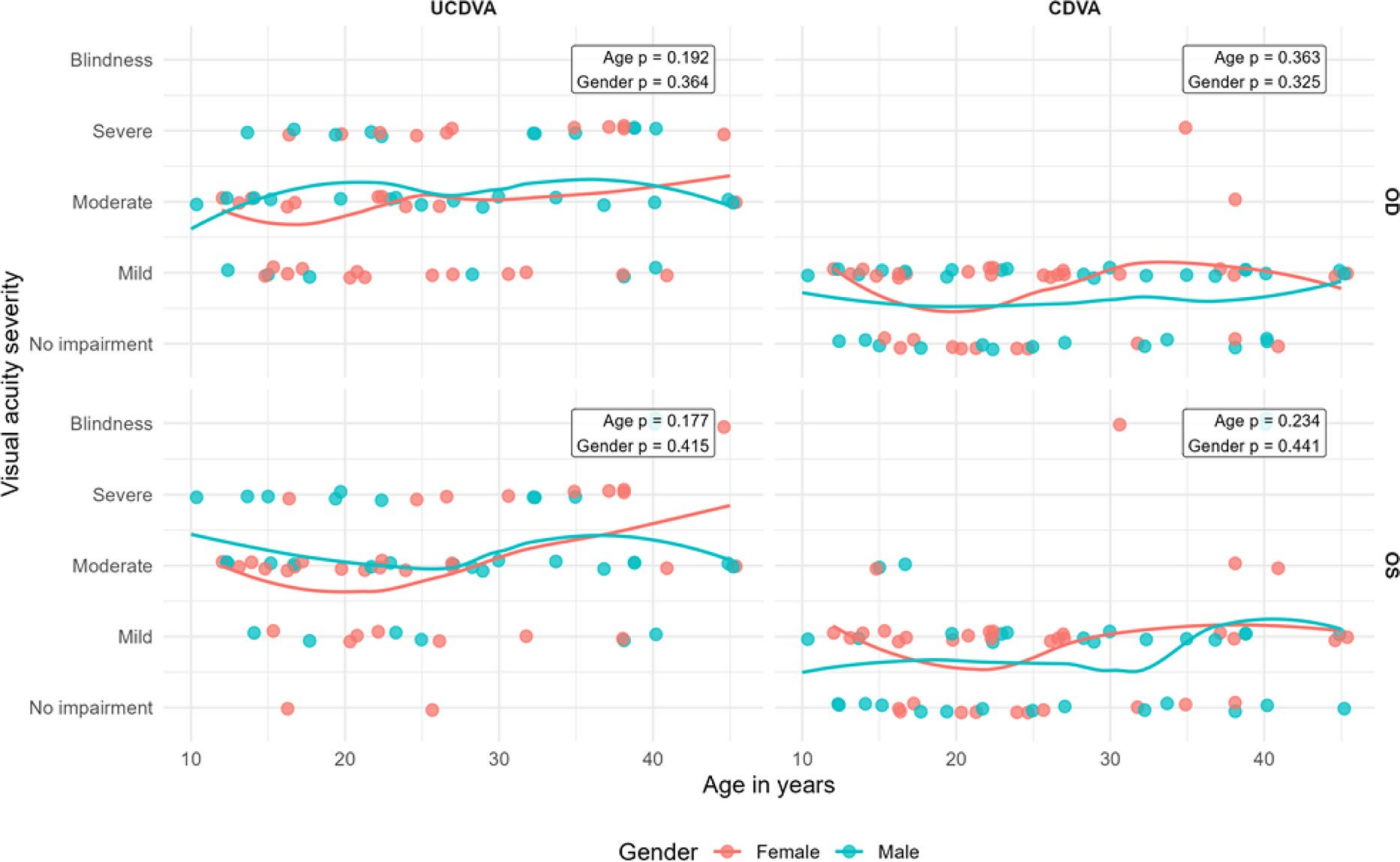
The distribution of UCDVA and CDVA severity categories by age, gender, and eye. *Key* OD* = Oculus dexter (Right eye), OS* = oculus Sinister (left eye), UCDVA^*^ = Uncorrected distance visual acuity

Ordinal logistic regression showed no evidence that age or gender influenced the severity of visual impairment based on either uncorrected or corrected distance visual acuity in either eye (all *p* > 0.05). Correction improved visual acuity overall, with fewer observations in the severe impairment categories under CDVA compared with UCDVA (Fig. 4).

### Distribution of uncorrected and best-corrected distance visual acuity (better eye)

**Fig. 5:**
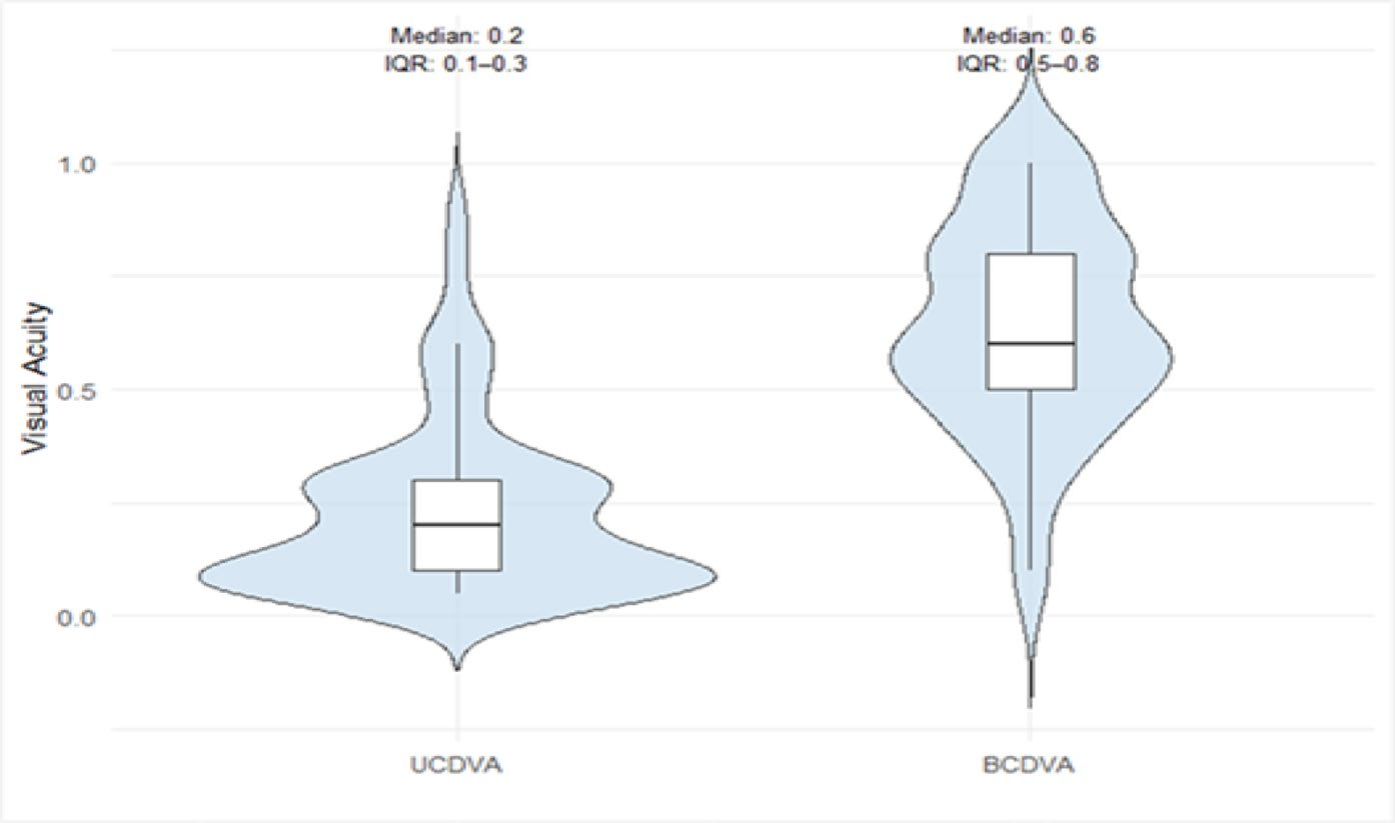
Distribution of uncorrected and best-corrected distance visual acuity (better eye)

Figure 5 shows the distribution of UCDVA and BCDVA in the study population. Median visual acuity improved from 0.2 (IQR: 0.1–0.3) for UCDVA to 0.6 (IQR: 0.5–0.8) for BCDVA. The entire visual acuity distribution shifted toward higher values after correction, demonstrating substantial visual improvement with refractive correction.

### Uncorrected visual acuity (UCDVA) relationships

**Fig. 6:**
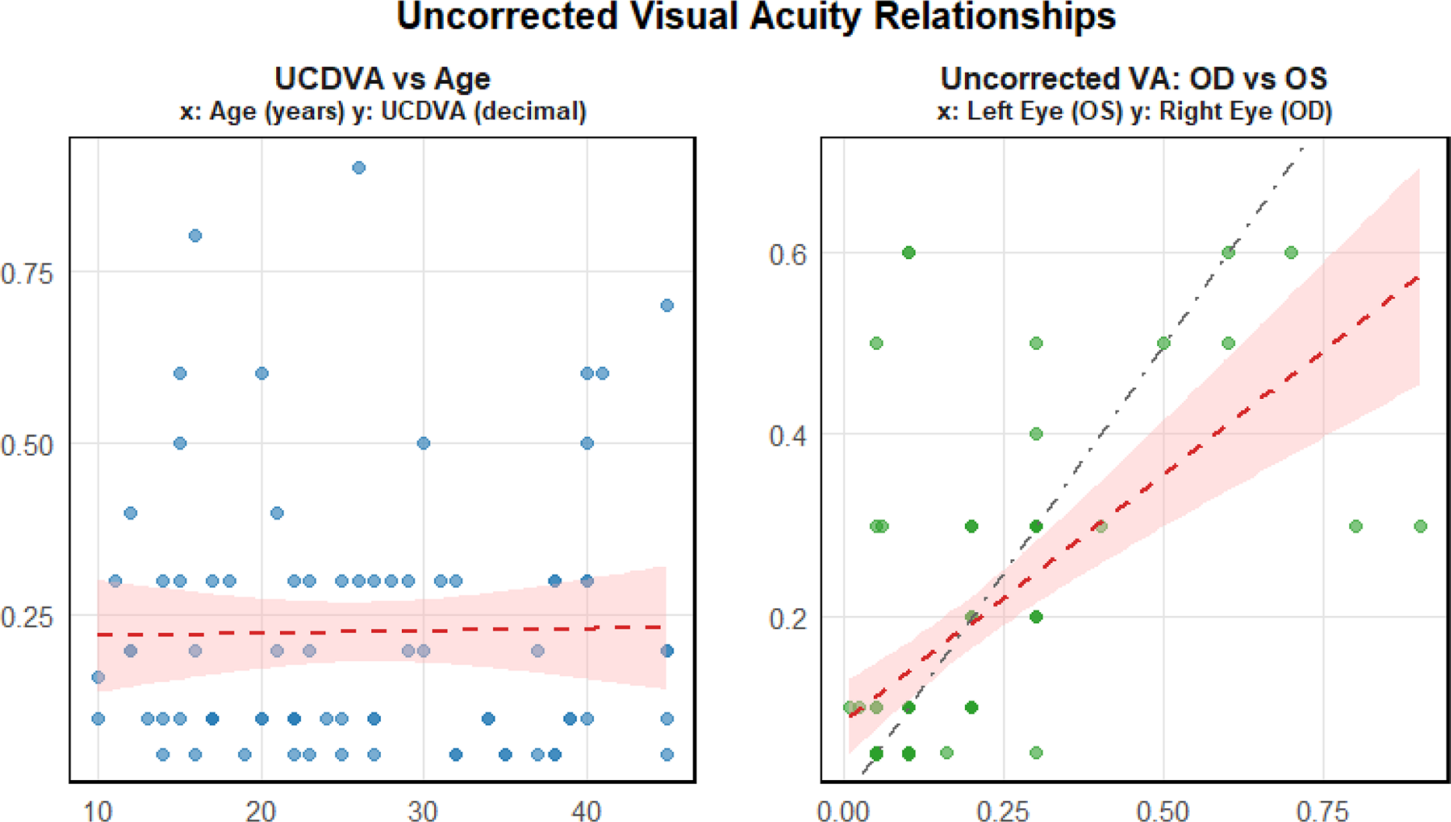
Uncorrected visual acuity

Figure 6 presents scatterplots illustrating relationships in uncorrected distance visual acuity (UCDVA). The left panel shows a weak association between age and UCDVA, with substantial variability across the age range. The right panel demonstrates a positive relationship between left-eye and right-eye UCDVA, with many observations distributed around the line of equality, indicating general inter-eye symmetry in uncorrected visual acuity, although some degree of asymmetry was present.

### Factors associated with keratoconus (KC)

Two multivariable logistic regression models were fitted to identify independent predictors of keratoconus (KC). Model A included region as a contextual variable, whereas Model B replaced region with facility. Both models adjusted for age group, gender and cylindrical component (left eye, OS).

#### Model A: Region-adjusted model

In the region-adjusted model (Model A), age group and gender were not independently associated with keratoconus after adjustment for other covariates (all *p* > 0.05). Compared with participants from the Central Region, those from the Southern Region had significantly higher odds of keratoconus (aOR = 7.02; 95% CI 1.67–29.4; *p* < 0.05), whereas the association for the Northern Region did not reach statistical significance (aOR = 7.78; 95% CI 0.41–148.9; *p* = 0.17). The cylindrical component of the left eye (OS) remained a strong independent predictor of keratoconus (aOR = 0.28; 95% CI 0.12–0.66; *p* = 0.003). Because cylindrical power was recorded in negative cylinder notation, more negative cylinder values were associated with increased odds of keratoconus. The model demonstrated excellent discrimination (AUC = 0.883), indicating good ability to distinguish keratoconus from non-keratoconus cases (Table 6).

**Table 6:** Multivariable logistic regression models for predictors of keratoconus

Variable	Category	Model A (*Region-adjusted)* (aOR [95% CI])	*p*-value	Model B (*Facility-adjusted)* (aOR [95% CI])	*p*-value
Age group	< 18	Ref		Ref	
	18–35	1.52 (0.30–7.64)	0.61	1.53 (0.31–7.62)	0.61
	36–45	0.44 (0.06–3.05)	0.41	0.51 (0.07–3.56)	0.50
Gender	Female	Ref		Ref	
	Male	1.61 (0.40–6.50)	0.51	1.51 (0.37–6.14)	0.56
Region	Central	Ref		Ref	
	Northern	7.78 (0.41–148.9)	0.17	–	–
	Southern	7.02 (1.67–29.4)	**	–	–
Facility	KCH	Ref		Ref	
	MCH	–	–	7.83 (0.41–150.8)	0.17
	QECH	–	–	5.21 (0.91–29.9)	0.064
	ZCH	–	–	9.51 (1.72–52.6)	**
Cylinder component (OS)	Per 1D increase (–ve)	0.28 (0.12 – 0.66)	**	0.28 (0.12–0.66)	**
Model performance	AUC (95% approx.)	0.883	–	0.882	–

*aOR* Adjusted odds ratio, *CI* Confidence interval

^*^p-value < 0.05; **p-value < 0.01;***p-value < 0.001

#### ROC comparison between region and facility adjusted models

**Fig. 7:**
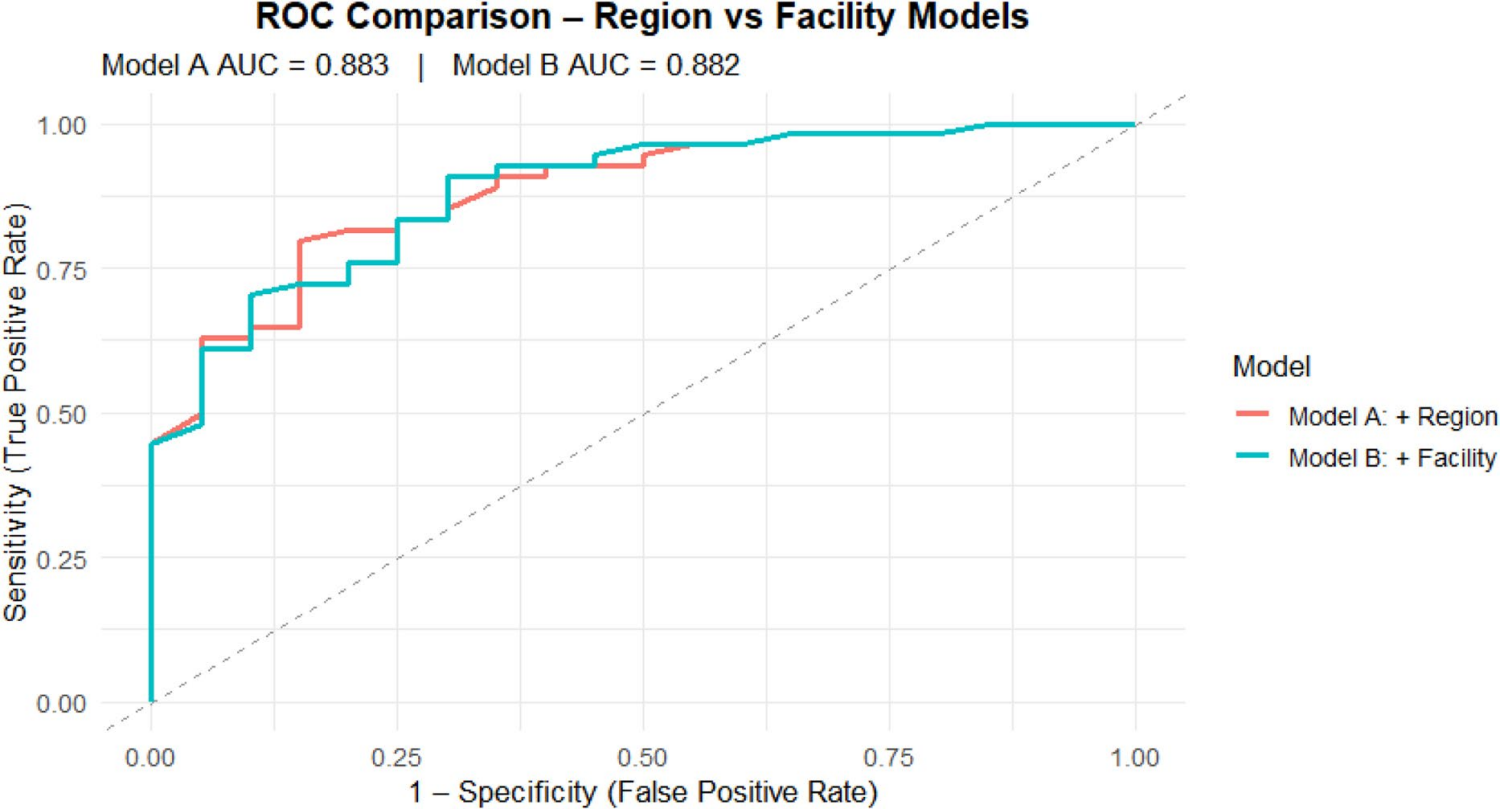
Model performance and sensitivity analysis

ROC analysis demonstrated excellent discrimination for both models, with virtually identical performance (AUC = 0.883 and 0.882, respectively; Fig. 7).

#### Model B: Facility-adjusted model (sensitivity analysis)

When region was replaced by facility, the findings remained broadly consistent. Age group and gender were not independently associated with keratoconus (all *p* > 0.05). Participants attending ZCH had significantly higher odds of keratoconus compared with those attending KCH (aOR = 9.51; 95% CI 1.72–52.6; *p* = 0.010), whereas QECH showed a borderline association (aOR = 5.21; 95% CI 0.91–29.9; *p* = 0.064). The cylindrical component (OS) remained an independent predictor of keratoconus (aOR = 0.28; 95% CI 0.12–0.66; *p* = 0.003). Model discrimination remained excellent (AUC = 0.882), supporting the robustness of the findings across geographic and facility-adjusted models (Table 6).

#### Events-per-variable assessment

**Fig. 8:**
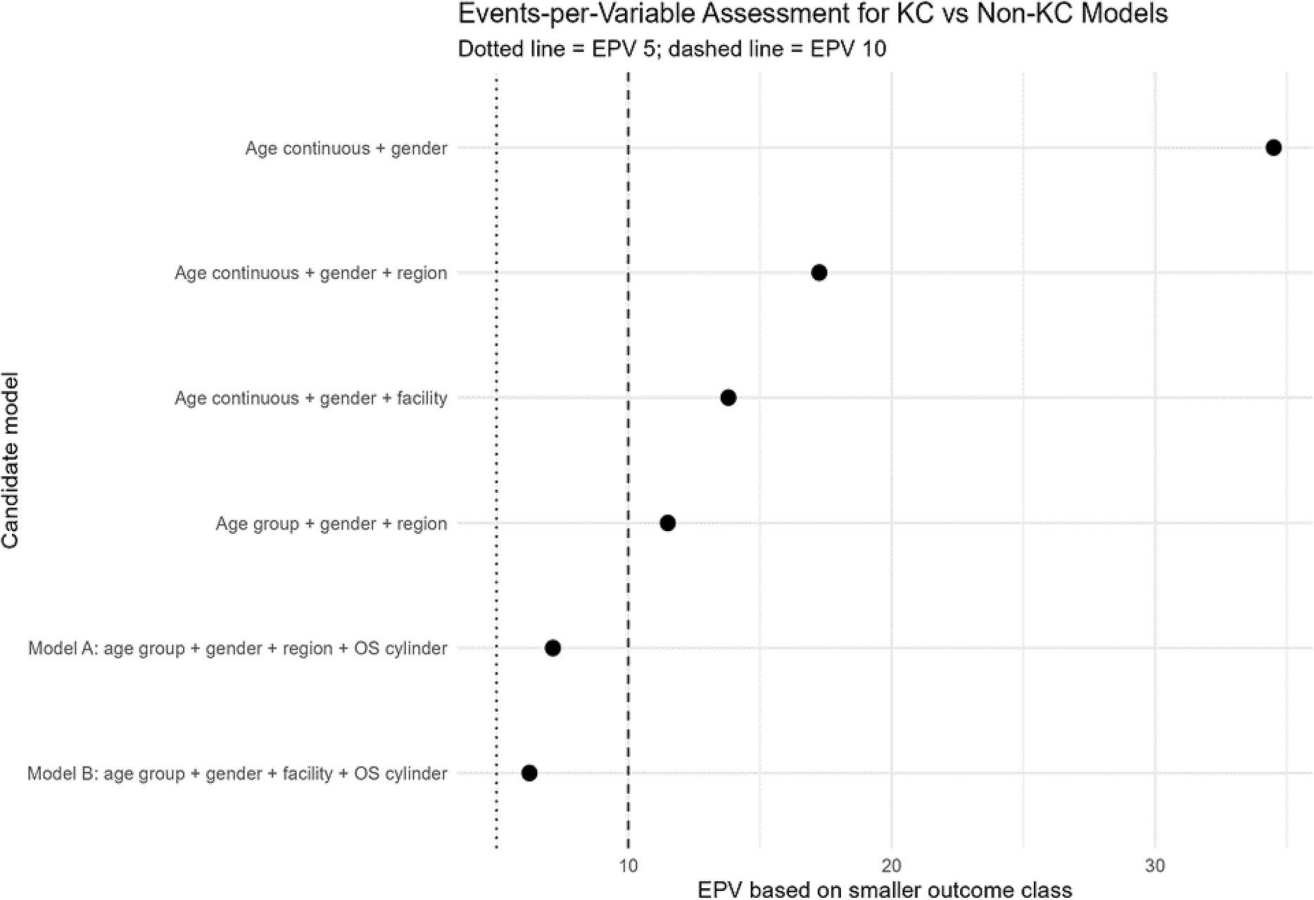
Events-per-variable assessment for KC vs Non KC models

EPV values ranged from 6.0 to 34.0 across candidate logistic regression models (Fig. 8). The highest EPV was observed for the age continuous + gender model (34.0), whereas the lowest was observed for Model B (age group + gender + facility + OS cylinder; EPV = 6.0). Three models achieved EPV values ≥ 10, while the remaining two models had EPV values between 5 and 10.

#### Conventional and Firth penalized logistic regression estimates for predictors of keratoconus

**Fig. 9:**
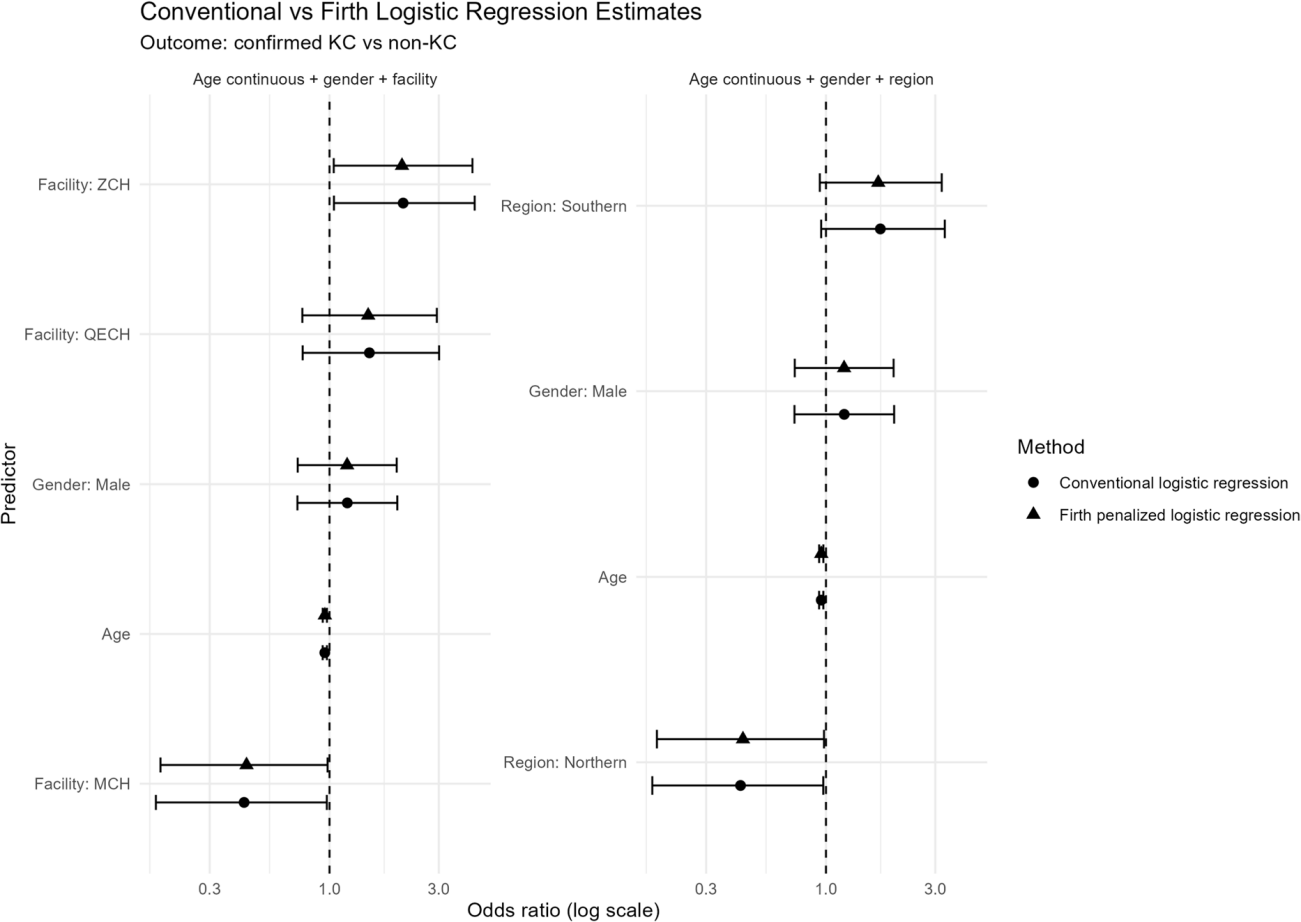
Comparison of conventional and Firth penalized logistic regression estimates for predictors of keratoconus

Firth penalized logistic regression yielded odds ratio estimates that were generally consistent with the conventional logistic regression models (Fig. 9). Although some coefficients were modestly attenuated, the overall pattern of associations remained unchanged, indicating that sparse-data bias was unlikely to have materially influenced the study conclusions.

## Discussion

### Proportion of keratoconus

This study provides the first multi-centre assessment of the facility-based proportion and clinical characteristics of keratoconus among patients attending tertiary referral refraction clinics in Malawi. Using records from the country’s four major referral eye hospitals, the study found a facility-based keratoconus proportion of 5.7% (95% CI 4.4–7.2%) (Table 3). This is comparable to the reports from other hospital-based studies in sub-Saharan Africa [13, 22, 23], but higher than many population-based estimates reported globally [5, 24, 25]. The comparisons across studies should, however, be interpreted cautiously because estimates are highly influenced by differences in sampling frames, referral pathways, case definitions, and availability of corneal imaging technologies. The relatively high proportion observed in this study likely reflects the concentration of complex refractive and corneal cases within tertiary referral centres rather than the true occurrence of keratoconus in the general Malawian population. Although this estimate cannot be interpreted as population prevalence because the study was restricted to patients seeking eye care at referral facilities, it nevertheless demonstrates that keratoconus represents an important clinical burden within specialised refractive services in Malawi. The finding contributes valuable evidence from a setting where epidemiological data on keratoconus are largely unavailable and where access to advanced diagnostic technologies remains limited.

### Demographic and regional characteristics by keratoconus status

The highest proportion of keratoconus was observed among individuals aged 18–35 years, consistent with the well established natural history of the disease. Keratoconus typically manifests during adolescence and progresses through early adulthood before stabilising later in life [11–16, 26]. The concentration of cases within younger age groups highlights the importance of early detection and intervention, particularly because visual impairment during economically productive years can have substantial social and economic consequences [27]. The lower proportion observed among participants aged 36–45 years may therefore reflect disease stabilisation, reduced presentation to refractive clinics, or cohort differences in health-seeking behaviour. No significant gender differences were observed (Table 3), supporting findings from several previous studies that have reported similar distributions between males and females [11–16], although sex-related patterns remain inconsistent across the global literature [28].

Significant geographic (*p* = 0.010) and facility-level (*p* = 0.002) variation was observed in this study (Table 3). Higher odds observed in the Southern Region and at Zomba Central Hospital may reflect differential access to tertiary eye-care services, referral concentration of complex corneal cases, or regional variation in clinician awareness and diagnostic practices. The absence of population-based data prevents determination of whether these differences represent true epidemiological variation or healthcare system effects. Similar findings in both the region-adjusted and facility-adjusted models support the consistency of these associations (Fig. 7 and Table 6). However, the wide confidence intervals indicate limited precision, and these differences should be interpreted cautiously. The observed variation may reflect differences in referral pathways, service accessibility, clinician awareness or record completeness. Further prospective studies are needed to determine the underlying causes of these patterns.

### Clinical and diagnostic characteristics

#### Health information infrastructure and research capacity

Of the 1,400 targeted records, 1,180 were successfully retrieved from all four national referral eye hospitals, providing a uniquely broad assessment of keratoconus within Malawi’s tertiary eye-care system. Because record retrieval varied across facilities, it was not possible to determine whether missing records differed systematically with respect to patient characteristics or keratoconus status. Consequently, some degree of selection bias cannot be excluded and the reported proportion may have been either under- or overestimated. Beyond their implications for research, these findings highlight the importance of strengthening routine ophthalmic documentation and implementing electronic medical records to support continuity of care, disease surveillance, and service planning.

#### Case definition, ascertainment and diagnostic limitations

The clinical presentation observed in this cohort was characteristic of keratoconus, reflecting the visual consequences of progressive corneal ectasia and irregular astigmatism. However, the diagnostic approach available within participating facilities relied predominantly on retinoscopy, slit-lamp examination, and keratometry, highlighting the realities of keratoconus detection in resource-constrained settings [21, 22]. However, the limited use of pachymetry and absence of advanced corneal imaging [29–31] likely reduced the detection of mild or subclinical keratoconus, potentially leading to underestimation of the facility-based proportion. Conversely, reliance on clinical judgement and variable documentation may have introduced some degree of misclassification. To evaluate this possibility, a sensitivity analysis restricted to cases with strong clinical evidence, including documented keratometry values and disease staging, was performed [Fig. 1]. The consistency of findings between the primary and sensitivity analyses supports the robustness of the overall conclusions, although some residual misclassification cannot be excluded.

#### Inter-eye symmetry and functional expression of keratoconus

The high degree of bilateral symmetry observed in keratometric and refractive parameters is consistent with the recognised bilateral nature of keratoconus, despite its often asymmetric clinical expression (Table 4, Figs. 2 and 3) [12, 13, 29–31]. Although steep K, flat K, spherical power, and cylindrical power did not differ significantly between eyes, significant inter-eye differences in BCVA and astigmatic axis were identified. This apparent dissociation between structural and functional measures suggests that visual impairment in keratoconus is not determined solely by corneal curvature. Rather, it may reflect the combined influence of higher-order aberrations, irregular astigmatism, corneal scarring, and other optical disturbances [1–3] that are not fully captured by conventional keratometric measurements. These findings underscore the importance of comprehensive bilateral assessment, as similar keratometric profiles may mask clinically meaningful differences in visual function and disease impact.

#### Disease severity classification and interpretation

Interpretation of disease severity requires caution because historical clinical records did not routinely include topography- or tomography-derived Kmax measurements, the contemporary standard for assessing keratoconus progression [30, 31]. Consequently, severity classification was based on the steepest available manual or automated keratometry readings (Fig. 1), which provide a pragmatic surrogate for corneal steepening in resource-constrained settings [21, 22]. Although this approach enabled severity stratification using routinely collected clinical data, it is unlikely to capture the full structural complexity of keratoconus. The original CLEK classification incorporates multiple clinical parameters and was not intended as a simple keratometry-based staging system [16]. Therefore, the severity categories used in this study should be interpreted as operational classifications rather than formal CLEK stages. While some degree of stage misclassification, particularly among borderline cases, cannot be excluded, the approach reflects real-world diagnostic practice in many low-resource eye-care settings and provides valuable insight into the spectrum of disease encountered in routine clinical care.

#### Visual impairment and rehabilitative care

Visual acuity findings revealed a substantial burden of functional visual impairment among patients with keratoconus (Figs. 4, 5 and 6). Although refractive correction resulted in clinically meaningful improvement, visual outcomes remained suboptimal for many patients, indicating that conventional refractive correction alone was insufficient to fully restore visual function. This likely reflects the concentration of moderate-to-advanced disease within tertiary referral services, where irregular astigmatism and higher-order aberrations often limit the effectiveness of spectacle correction [32]. The low utilisation of rigid gas-permeable contact lenses, despite their established role in visual rehabilitation [14, 15, 22, 26, 33, 34], further suggests constrained access to specialised keratoconus care. Moreover, best-corrected visual acuity measurements largely reflected spectacle-based rather than contact lens assisted correction, potentially underestimating achievable visual outcomes [34]. Collectively, these findings highlight both the visual burden of keratoconus at presentation and gaps in the availability of specialised rehabilitative and surgical services [35, 36] services, underscoring the need to strengthen access to contact lens fitting, early diagnosis, and disease modifying interventions to optimise long-term visual outcomes.

### Multivariable analysis and predictors of keratoconus

Multivariable analyses identified cylindrical refractive error as the strongest independent correlate of keratoconus, while age and gender were not independently associated with disease after adjustment (Table 6). The consistent association between cylindrical power and keratoconus across alternative model specifications underscores the central role of irregular astigmatism in the clinical expression of the disease and highlights the diagnostic value of careful refraction in settings where advanced corneal imaging is not routinely available [22–24, 26, 34]. Although both models demonstrated excellent discrimination (AUC ≈ 0.88), the relatively small number of keratoconus cases (n = 67) resulted in wide confidence intervals for some estimates and increased the risk of overfitting in more complex models. To assess the potential influence of sparse-data bias, a Firth penalized logistic regression sensitivity analysis was performed (Figs. 8 and 9). The close agreement between penalized and conventional estimates suggests that sparse-data bias did not materially affect the observed associations. Nevertheless, the limited precision of some geographic and facility-level estimates warrants cautious interpretation, and these findings should be regarded as exploratory and hypothesis-generating. Larger prospective studies are needed to confirm these associations and provide more precise estimates of risk.

Collectively, these findings suggest that keratoconus represents an important but potentially under-recognised cause of visual impairment within Malawi’s tertiary eye-care system. The combination of delayed presentation, limited access to advanced diagnostic technologies, and constrained rehabilitative services highlights opportunities for strengthening keratoconus detection and management pathways. Improving access to corneal imaging, specialised contact lens services, and robust clinical information systems may facilitate earlier diagnosis and improve visual outcomes.

### Strengths, limitations and implications

This study provides the first multicentre assessment of the facility-based proportion, clinical characteristics, and management of keratoconus among patients attending tertiary referral eye-care services in Malawi. By including records from all four national referral eye hospitals, it offers important baseline evidence from a setting where epidemiological data on keratoconus remain scarce. A methodological strength of this study was the use of Firth penalized logistic regression as a sensitivity analysis to evaluate potential sparse-data bias arising from the relatively small number of keratoconus cases. The findings therefore, highlight keratoconus as a significant contributor to visual impairment within specialised refractive services and underscore the challenges associated with delayed presentation, limited diagnostic capacity, and restricted access to specialised visual rehabilitation.

Several limitations should be considered when interpreting these findings. First, the retrospective design relied on the completeness and accuracy of routinely collected clinical records. Second, only 1,180 of the targeted 1,400 records were retrieved because of missing files, incomplete archiving systems, and variable record-storage practices across facilities, potentially introducing selection bias. Third, the study was facility-based and therefore the findings cannot be generalised to the wider Malawian population. Fourth, case ascertainment and severity classification were constrained by limited access to corneal imaging technologies, creating the potential for diagnostic and staging misclassification. Finally, the relatively small number of keratoconus cases limited the precision of multivariable analyses and necessitates cautious interpretation of adjusted associations.

## Conclusion

This study provides the first multicentre assessment of keratoconus within Malawi’s tertiary eye-care system and demonstrates that keratoconus constitutes a substantial clinical burden among patients attending referral refraction services. The disease predominantly affected younger individuals and was associated with significant visual impairment, although refractive correction produced meaningful visual improvement. Cylindrical refractive error emerged as the strongest clinical correlate of keratoconus, underscoring the continued importance of careful refraction, retinoscopy, and clinical examination where advanced corneal imaging is unavailable. Although limitations related to retrospective data collection, incomplete record retrieval, and constrained diagnostic infrastructure warrant cautious interpretation, the consistency of findings across sensitivity analyses supports the overall validity of the results. Strengthening early detection, diagnostic capacity, specialised rehabilitation services, and clinical information systems may improve the identification and management of keratoconus in Malawi and similar resource-constrained settings.

## Recommendations

1. **Improve clinical documentation and health information systems**, including the implementation of electronic medical records, to strengthen continuity of care, disease surveillance, and research capacity.
2. **Develop national multi-disciplinary, multi-sectoral diagnosis and management algorithm** to support standardised diagnosis, classification, and management of keratoconus across all levels of eye-care services in Malawi.
3. **Strengthen early detection and referral pathways** through routine keratoconus screening of young patients presenting with progressive astigmatism, reduced visual acuity, or suspicious retinoscopic findings.
4. **Expand access to diagnostic technologies**, including corneal topography, tomography, and pachymetry, to improve detection of early and subclinical disease and support more accurate severity assessment of KC in Malawi.
5. **Enhance keratoconus management services in all central hospitals across Malawi** by increasing availability of rigid gas-permeable and specialty contact lenses, as well as access to corneal cross-linking and other vision-preserving interventions.
6. **Conduct prospective, population-based studies** to establish the true epidemiology of keratoconus in Malawi and validate the associations identified in this facility-based analysis.

## Data Availability

No datasets were generated or analysed during the current study.

## References

[1] Singh RB, Koh S, Sharma N, Woreta FA, Hafezi F, Dua HS, Jhanji V (2024) Keratoconus. Nat Rev Dis Primers 10(1):81 39448666 10.1038/s41572-024-00565-3

[2] Cheung IM, Angelo L, Gokul A, Ziaei M (2025) Non-genetic risk factors for keratoconus and its progression. Clin Exp Optom 108(6):648–656 39762118 10.1080/08164622.2024.2443454

[3] Almusawi LA, Hamied FM (2021) Risk factors for development of keratoconus: a matched pair case-control study. Clin Ophthalmol. 10.2147/opth.s248724 34429579 10.2147/OPTH.S248724 PMC8378899

[4] Gordon-Shaag A, Millodot M, Shneor E, Liu Y (2015) The genetic and environmental factors for keratoconus. BioMed Res Int. 10.1155/2015/795738 26075261 10.1155/2015/795738 PMC4449900

[5] Hashemi H, Beiranvand A, Khabazkhoob M, Asgari S, Emamian MH, Shariati M, Fotouhi A (2013) Prevalence of keratoconus in a population-based study in Shahroud. Cornea 32(11):1441–1445 24042484 10.1097/ICO.0b013e3182a0d014

[6] Hashemi H, Yekta A, Khabazkhoob M (2015) Effect of keratoconus grades on repeatability of keratometry readings: comparison of 5 devices. J Cataract Refract Surg 41(5):1065–1072 26049838 10.1016/j.jcrs.2014.08.043

[7] Sahebjada S, Al-Mahrouqi HH, Moshegov S, Panchatcharam SM, Chan E, Daniell M, Baird PN (2021) Eye rubbing in the aetiology of keratoconus: a systematic review and meta-analysis. Graefes Arch Clin Exp Ophthalmol 259(8):2057–2067 33484296 10.1007/s00417-021-05081-8

[8] Deshmukh R, Ong ZZ, Rampat R, Alió del Barrio JL, Barua A, Ang M, Mehta JS, Said DG, Dua HS, Ambrósio R Jr, Ting DS (2023) Management of keratoconus: an updated review. Front Med 10:1212314 10.3389/fmed.2023.1212314 PMC10318194 37409272

[9] Gokul A, Patel DV, Watters GA, McGhee CN (2017) The natural history of corneal topographic progression of keratoconus after age 30 years in non-contact lens wearers. Br J Ophthalmol 101(6):839–844 27729309 10.1136/bjophthalmol-2016-308682

[10] Gomes JA, Tan D, Rapuano CJ, Belin MW, Ambrósio R Jr, Guell JL, Malecaze F, Nishida K, Sangwan VS (2015) Global consensus on keratoconus and ectatic diseases. Cornea 34(4):359–369 25738235 10.1097/ICO.0000000000000408

[11] Millodot M, Ortenberg I, Lahav-Yacouel K, Behrman S (2016) Effect of ageing on keratoconic corneas. J Optom 9(2):72–77 26142151 10.1016/j.optom.2015.05.001 PMC4812003

[12] Rabinowitz YS, Galvis V, Tello A, Rueda D, García JD (2021) Genetics vs chronic corneal mechanical trauma in the etiology of keratoconus. Exp Eye Res 202:108328 33172608 10.1016/j.exer.2020.108328

[13] Akowuah PK, Kobia-Acquah E, Donkor R, Adjei-Anang J, Ankamah-Lomotey S (2021) Keratoconus in Africa: a systematic review and meta-analysis. Ophthalmic Physiol Opt 41(4):736–747 33860963 10.1111/opo.12825

[14] Barbara R, Turnbull AM, Malem A, Anderson DF, Hossain P, Konstantopoulos A, Barbara A (2018) Epidemiology of keratoconus. Controversies in the management of keratoconus. Springer International Publishing, Cham, pp 1–16

[15] Aldayel AA, Alwael HM, Alshathri RM, Alnasser HA, Alfawzan LA, Alfawzan L (2022) A comparison between cross-linking protocols in patients with progressive keratoconus: a review. Cureus 14(11):e31029 36475196 10.7759/cureus.31029 PMC9718644

[16] Gideon Abou Said A, Piñero DP, Shneor E (2023) Revisiting the oil droplet sign in keratoconus: utility for early keratoconus diagnosis and screening. Ophthalmic Physiol Opt 43(1):83–92 36394095 10.1111/opo.13066 PMC10099609

[17] Wagner H, Barr JT, Zadnik K, Collaborative Longitudinal Evaluation of Keratoconus (CLEK) Study Group (2007) Collaborative longitudinal evaluation of keratoconus (CLEK) study: methods and findings to date. Contact Lens Anterior Eye 30(4):223–232 17481941 10.1016/j.clae.2007.03.001 PMC3966142

[18] Giannaccare G, Murano G, Carnevali A, Yu AC, Vaccaro S, Scuteri G, Maltese L, Scorcia V (2021) Comparison of Amsler–Krumeich and Sandali classifications for staging eyes with keratoconus. Appl Sci 11(9):4007

[19] Belin MW, Kundu G, Shetty N, Gupta K, Mullick R, Thakur P (2020) ABCD: a new classification for keratoconus. Indian J Ophthalmol 68(12):2831–2834 33229658 10.4103/ijo.IJO_2078_20 PMC7856970

[20] Mahasneh S, Abiad BH, Cavanagh HD (2018) Comparison of visual outcomes of deep anterior lamellar keratoplasty versus penetrating keratoplasty in patients with keratoconus. In: 2018 ASCRS ASOA Annual Meeting. ASCRS

[21] Barbara A, Barbara R (2013) Intacs intracorneal ring segments complications in patients suffering from keratoconus. Int J Keratoconus Ectatic Corneal Dis 2(3):121

[22] Gcabashe NM, Moodley VR, Hansraj R (2023) Prevalence and clinical profile of keratoconus in patients presenting at a provincial hospital in KwaZulu-Natal, South Africa: a case study. J Public Health Afr 14(9):2356 37942062 10.4081/jphia.2023.2356 PMC10628798

[23] Kobia-Acquah E, Senanu EN, Antwi-Adjei EK, Appiah DP, Kumah DB, Abdul-Kabir M, Donkor R (2022) Prevalence of keratoconus in Ghana: a hospital-based study of tertiary eye care facilities. Eur J Ophthalmol 32(6):3185–3194 35818728 10.1177/11206721221113197

[24] Hashemi H, Heydarian S, Hooshmand E, Saatchi M, Yekta A, Aghamirsalim M, Valadkhan M, Mortazavi M, Hashemi A, Khabazkhoob M (2020) The prevalence and risk factors for keratoconus: a systematic review and meta-analysis. Cornea 39(2):263–270 31498247 10.1097/ICO.0000000000002150

[25] Sriranganathan A, Chan CC, Dhillon J, Felfeli T (2022) Global incidence and prevalence of keratoconus: a systematic review and meta-analysis. Cornea 13:10–97 10.1097/ICO.0000000000003973 40833011

[26] Rashid ZA, Millodot M, Evans KS (2016) Characteristics of keratoconic patients attending a specialist contact lens clinic in Kenya. Middle East Afr J Ophthalmol 23(4):283–287 27994389 10.4103/0974-9233.194074 PMC5141619

[27] Marques AP, Ramke J, Cairns J, Butt T, Zhang JH, Muirhead D, Jones I, Tong BA, Swenor BK, Faal H, Bourne RR (2021) Global economic productivity losses from vision impairment and blindness. EClinicalMedicine. 10.1016/j.eclinm.2021.100852 33997744 10.1016/j.eclinm.2021.100852 PMC8093883

[28] Shah ZA, Purohit DM, Danayak PM, Patel JD, Purohit SM (2024) Keratoconus presentation with respect to age, gender, and severity in Western India. J Clin Ophthalmol Res 12(2):96–100

[29] Safir M, Nitzan I, Hanina Y, Heller D, Mimouni M, Sorkin N (2025) Keratoconus prevalence in astigmatic adolescents: findings from a nationwide screening setting. Eye (Lond) 39(16):2958–2962 40968150 10.1038/s41433-025-03995-9 PMC12583458

[30] Hashemi H, Khabazkhoob M, Fotouhi A (2013) Topographic keratoconus is not rare in an Iranian population: the Tehran eye study. Ophthalmic Epidemiol 20(6):385–391 24168025 10.3109/09286586.2013.848458

[31] Kanclerz P, Khoramnia R, Wang X (2021) Current developments in corneal topography and tomography. Diagnostics (Basel) 11(8):1466 34441401 10.3390/diagnostics11081466 PMC8392046

[32] Azizi E (2024) Prescription of spectacles in keratoconus. Keratoconus. CRC Press, pp 74–79

[33] Moschos MM, Nitoda E, Georgoudis P, Balidis M, Karageorgiadis E, Kozeis N (2017) Contact lenses for keratoconus-current practice. Open Ophthalmol J 11:241 28932340 10.2174/1874364101711010241 PMC5585463

[34] Nkoana PM, Moodley VR, Mashige KP (2023) Keratoconic patient profile and management at public sector facilities in South Africa. Afr Vision Eye Health 82(1):780

[35] Mandathara PS, Stapleton FJ, Willcox MD (2017) Outcome of keratoconus management: review of the past 20 years’ contemporary treatment modalities. Eye Contact Lens 43(3):141–154 27171132 10.1097/ICL.0000000000000270

[36] Baenninger PB, Bodmer NS, Bachmann LM, Iselin K, Kaufmann C, Belin MW, Thiel MA (2020) Keratoconus characteristics used in randomized trials of surgical interventions—a systematic review. Cornea 39(5):615–620 31738244 10.1097/ICO.0000000000002202

